# Effects of physical disability and widowhood on the survival of centenarians: a 7-year follow-up of CHCCS centenarians

**DOI:** 10.3389/fragi.2026.1764198

**Published:** 2026-02-24

**Authors:** Songmei Han, Dongxu Zhao, Jianbo Wu, Mingzhi Shen, Fei Hua

**Affiliations:** 1 Department of Endocrinology, The Third Affiliated Hospital of Soochow University, Changzhou, China; 2 Department of General Medicine, Hainan Hospital of Chinese People’s Liberation Army General Hospital, Sanya, China; 3 Department of General Surgery, The Second Naval Hospital of Southern Theater Command of People’s Liberation Army, Sanya, China

**Keywords:** activity of daily life, centenarian, physical disability, survival, widow

## Abstract

**Background:**

Centenarians, being at the end of their life span, are particularly vulnerable to various health risks. Multiple factors can influence their survival and targeted intervention on these factors may promote healthy aging.

**Purpose:**

This study aims to explore the effect of physical disability and widowhood on the survival of centenarians.

**Methods:**

Based on the China Hainan Centenarian Cohort Study (CHCCS), this study followed 787 centenarians for 7 years. Data were collected using formal designed questionnaire, physical examination and experimental tests. Questionnaire contains information including geographical data, cognitive and physical function. All the participants were followed-up annually. The endpoint of the follow-up was death or the end of the study. Cox regression analysis was conducted to identify survival-related factors, followed by stratified analysis according to their marital status.

**Results:**

Out of the 787 centenarians, only 382 survived after 7 years, resulting in a mortality rate of 51.46%. Multivariate Cox regression analysis showed that activities of daily living (ADL) (for ADL<60 vs. ADL≥90: HR = 1.933, 95% CI: 1.411–2.648, *p* < 0.001; for 60≤ADL<90 vs. ADL≥90: HR = 1.438, 95% CI: 1.084–1.907, *p* = 0.012) was significant factors affecting survival. Stratified analysis based on marital status showed that physical disability was an influence factor of survival in widowed (for ADL<60 vs. ADL≥90, HR = 2.020, 95% CI: 1.450–2.814, *p* < 0.001; for 60≤ADL<90 vs. ADL≥90, HR = 1.493, 95% CI: 1.108–2.011, *p* = 0.008) centenarians.

**Conclusion:**

Physical disability and widowhood were important predictors of survival among centenarians. Adequate external assistance should be provided to the disabled and widowed centenarians to enhance their quality of life and survival prospects.

## 1 Introduction

Due to the physiological limitations, the proportion of centenarians within the population remains very low, and research on this unique group is relatively scarce. While most people aspire to live as long as possible, advancements in material resources and medical science have increased this possibility (Li et al., 2024). However, with the extension of life span, physical, cognitive, and multi-morbidity challenges inevitably accumulate (Robert and Fulop, 2014). Data from the Chinese Longitudinal Healthy Longevity Study (CLHLS) showed that activities of daily living (ADL) decreased by 0.8%–2.8% annually in individuals aged 80 years and older (Zeng et al., 2017). Compared with the general elderly population, centenarians exhibit a higher dependency on medical resources, social care, as well as associated care costs (Chen and Park, 2023). Understanding the influencing factors contributing to disability and mortality in this population is crucial, as targeted interventions can help reduce costs and improve the efficiency of limited social resources (Cruces-Salguero et al., 2022; Zhao et al., 2020).

Among the various challenges faced by centenarians, the decline in physical function is pronounced (Lepers et al., 2016). A modeling study (Zimmer et al., 2012) of individuals aged 80 and older revealed an increase in the number of people with impaired ADL as age progresses. This decline in physical function often places centenarians at greater risk of health deterioration and mortality (Mosfeldt et al., 2019). An analysis of 13666 individuals aged 65 and older from the CLHLS (2002–2014) showed that the hazard ratio (HR) for death among individuals with disabilities was 1.44 (95%CI: 1.48–1.90) compared with those without disabilities (Yang et al., 2021). The deteriorated physical function of centenarians also have a detrimental effect on their mental health. Therefore, this study proposes the following hypothese:

H1 Physical disability negatively influence the survival of centenarians.

Widowhood, which occurs at various stages of adulthood, significantly impacts both the physical and psychological wellbeing of those affected (Wang et al., 2024). The consequences can be both short-term and long-term. In the early stages of losing a partner, survivors may experience severe psychological and emotional distress (Sheftel et al., 2024). Over time, long-term widows often experience feelings of loneliness and lack of dependence (Yang, 2021; Yang and Gu, 2021). Previous studies have confirmed the relationship between widowhood and increased mortality (Barrenetxea et al., 2023; Blanner et al., 2020). A cross-sectional survey (Hsiao et al., 2021) in Taiwan showed that widowed individuals had a higher risk of death compared with the non-widowed individuals. Similarly, a study (Barrenetxea et al., 2023) based on 15,858 Singaporean Chinese aged 61–96 found that widowed adults had a higher mortality risk (HR = 1.18, 95% CI: 1.01–1.38). However, there is limited research on the impact of widowhood on the survival of centenarians, suggesting the need for greater attention to the health status and marital circumstances of centenarians. Therefore, this study proposes the following hypotheses:

H2 Widowhood negatively influence the survival of centenarians.

Previous studies (Ho and Hung, 2013) have highlighted the importance of ADL and widowhood separately as predictors of mortality risk among ordinary population. Physically disabled centenarians lack the ability to complete daily life. Spouses are the closest partners of centenarians and can provide the most ideal external assistance for disabled centenarians. The loss of external help given by partners may cause physically disabled centenarians fail to complete basic daily activities, which may increase the risk of death.Therefore, this study proposes the following hypothese:

H3 Widowhood modify the associations between ADL and mortality.

Gaining insights into this relationship will help inform corresponding policies and strategies to support the health of centenarians. Therefore, this study, based on the China Hainan Centenarian Cohort Study (CHCCS) (He et al., 2018), aims to investigate the impact of physical disability and widowhood on the survival of centenarians.

## 2 Materials and methods

### 2.1 Study design and participants

**FIGURE 1:**
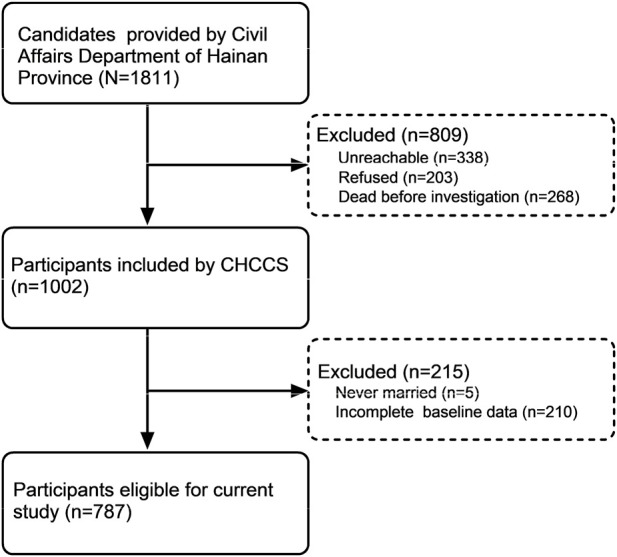
Flowchart of participant selection.

The data analyzed in the current study were derived from the CHCCS, the world’s largest cohort study of centenarians (He et al., 2018; He et al., 2017). The Civil Affairs Department of Hainan Province, China, provided a total of 1811 centenarians (age ≥100 years) as of 30 June 2014. At the conclusion of the investigation, 1473 centenarians were contactable, 268 had died, and 203 refused participation. Ultimately, 1002 centenarians participated in the CHCCS study. For this analysis, individuals who were never married or had incomplete baseline data were excluded, leaving 787 centenarians for the final analysis. (Figure 1).

Written informed consent was obtained from all participants before their inclusion in the study. This study was approved by the Ethics Committee of Hainan Hospital of PLA General Hospital (Sanya, Hainan; approval No. 301HNLL-2016-01).

### 2.2 Interview

Before the survey, a questionnaire for centenarians was designed in advance. Uniformly trained investigators conducted one-on-one interviews with the centenarians or their guardians and recorded the responses. The survey covered general demographic information, daily habits, disease history, and assessments of physical and cognitive function, among other topics. Dates of birth were checked to verify the registered age against the chronological age. Participants with no formal education were classified as Uneducated, while those with any formal education were classified as Educated. Smoking status was categorized as Yes for current or past daily smoking, and No for occasionally or no smoking. Drinking status was similarly categorized as Yes for current or past daily drinking, and No for occasional or no drinking.

### 2.3 Anthropometric measurements

Blood pressure was measured twice within a 15-min interval using an electronic sphygmomanometer, while the participant was in a quiet state in the morning. The systolic blood pressure (SBP) and diastolic blood pressure (DBP) were recorded as the average of the two measurements.

### 2.4 Laboratory tests

Fasting venous blood samples (5 mL) were collected by skilled nurses 15 min after the participants woke up in the morning and temporarily stored at 4 °C. The samples were tested at the Central Laboratory of our hospital for the following parameters: fasting blood glucose (FBG), total cholesterol (TC), and triglyceride (TG).

### 2.5 Cognitive function

Cognitive function was assessed using the Mini-Mental State Examination (MMSE) (Carpinelli Mazzi et al., 2020).The MMSE evaluates orientation in time, space and place, as well as delayed memory, immediate memory, language, attention, and calculation. The total MMSE score is the MMSE score was used as an indicator of cognitive function, with higher scores reflecting better cognitive performance.

### 2.6 Physical function

Physical function was assessed using the Barthel scale, represented by the ADL score (Wade and Collin, 1988). This scale evaluates the ability of the participants to independently perform 10 essential daily activities, including grooming, bathing, dressing, eating, and others. The total score is the ADL score, with a maximum possible score of 100. A score of 90–100 was classified as physically normal, a score below 90 as partially disabled, and a score below 60 as disabled.

### 2.7 Outcome definition

The date of the first investigation marked the beginning of the follow-up. The endpoint of the study was either the death of the centenarians or the date of 30 June 2021. Death dates were collected annually from the Civil Affairs Department of Hainan Province and were further verified by contacting the centenarians’ family members or guardians by telephone or home visits. The time interval between investigation and death was survival time, and was documented and analyzed in months.

### 2.8 Statistics analysis and model building

Data were processed using IBM SPSS Statistics v25.0 and R v4.1.3 software. Continuous variables with a normal distribution were expressed as means (standard deviation), and group comparisons were performed using the independent two-sample student t-test. Continuous variables with skewed distributions were presented as median (interquartile range), and inter-group comparisons were conducted using the non-parametric Mann-Whitney U rank sum test. Categorical data were expressed as frequencies (n, %), and the chi-square test was used for group comparison.

Univariate and multivariate Cox proportional hazard regression model was applied to identify factors influencing centenarian survival. Progressively adjusted covariates: Model 1 adjusted for no covariate; Model 2 adjusted for age and gender; Model 3 adjusted for age, gender, maritial status, residential type, No. of children, No. of diagnoses and MMSE. Furthermore, marital status was stratified to examine the impact of ADL on survival. Kaplan-Meier survival curves were generated, and differences between the curves were compared using the Log-rank test. The scatter plots of Schoenfeld residuals versus time and the Lowess smooth curves were drawed. Visualized inspecting of the Schoenfeld residuals over the time metric and the direction of the Lowess smooth curves were used to test the proportional hazards assumption in the Cox proportional hazards models. All tests were two-tailed, and a significance level α = 0.05 was used.

## 3 Results

### 3.1 Baseline characteristic of centenarians

A total of 787 centenarians were eligible for the final analysis. By the end of the follow-up period, 405 centenarians had died, resulting in a mortality rate of 51.46% (Table 1). Survivors were more likely to live alone (16.75% vs. 10.86%, *p* = 0.013). In addition, non-survivors had lower ADL scores compared to survivors [80 (55, 95) vs. 90 (65, 95), *p* = 0.001]. There were no statistical differences between groups in terms of age, gender, education, ethnicity, marital status, residential type, smoking, drinking, number of children, number of diagnoses, SBP, DBP, fasting blood glucose, total cholesterol, triglycerides or MMSE scores.

**TABLE 1:** Profiles of centenarians grouped by survival status.

Variables	All (n = 787)	Survivors (n = 382, 48.5%)	Non-survivors (n = 405, 51.8%)	*p*
Survival time, mean (SD), month	54 (15, 60)	60 (56, 71)	16 (5, 31)	0.000
Age, mean (SD), year	102 (101, 104)	102 (101, 104)	102 (101, 104)	0.852
Gender	​	​	​	0.322
Male	145 (18.4%)	65 (17.0%)	80 (19.8%)	​
Female	642 (81.6%)	317 (83.0%)	325 (80.2%)	​
Education	​	​	​	0.487
Educated	61 (7.8%)	27 (7.1%)	34 (8.4%)	​
Uneducated	726 (92.2%)	355 (92.9%)	371 (91.6%)	​
Ethnicity	​	​	​	0.773
Han	702 (89.2%)	342 (89.5%)	360 (88.9%)	​
Minority	85 (10.8%)	40 (10.5%)	45 (11.1%)	​
Marital status	​	​	​	0.771
Non-widowed	88 (11.2%)	44 (11.5%)	44 (10.9%)	​
Widowed	699 (88.8%)	338 (88.5%)	361 (89.1%)	​
Residential type	​	​	​	0.013
With family	670 (85.1%)	311 (81.4%)	359 (88.6%)	​
Live alone	108 (13.7%)	64 (16.8%)	44 (10.9%)	​
Smoke	​	​	​	0.168
Yes	69 (8.8%)	29 (7.6%)	40 (9.9%)	​
No	668 (84.9%)	339 (88.7%)	329 (81.2%)	​
Drink	​	​	​	0.364
Yes	80 (10.2%)	36 (9.4%)	44 (10.9%)	​
No	663 (84.2%)	334 (87.4%)	329 (81.2%)	​
No. of children, medium (IQR)	4 (3, 6)	4 (3, 6)	4 (3, 6)	0.925
No. of chronic diseases, medium (IQR)	1 (0, 2)	1 (0, 2)	1 (0, 2)	0.501
SBP, mean (SD), mmHg	151.56 (24.95)	151.61 (24.42)	151.51 (25.50)	0.956
DBP, mean (SD), mmHg	75.12 (13.03)	74.66 (12.25)	75.57 (13.76)	0.336
Fasting blood glucose, mean (SD), mmol/L	5.15 (1.47)	5.19 (1.43)	5.11 (1.50)	0.502
Total cholesterol, mean (SD), mmol/L	4.68 (1.04)	4.71 (1.01)	4.65 (1.07)	0.401
Triglyceride, mean (SD), mmol/L	1.18 (0.62)	1.21 (0.68)	1.15 (0.56)	0.180
MMSE	​	​	​	0.671
≤9	320 (40.7%)	154 (40.3%)	166 (41.0%)	​
10–20	203 (25.8%)	101 (26.4%)	102 (25.2%)	​
≥21	47 (6.0%)	20 (5.2%)	27 (6.7%)	​
ADL score, medium (IQR)	85 (60, 95)	90 (65, 95)	80 (55, 95)	0.001
ADL	​	​	​	0.002
60<	176 (22.4%)	69 (18.1%)	107 (26.4%)	​
60–89	235 (29.9%)	107 (28.0%)	128 (31.6%)	​
≥90	347 (44.1%)	190 (49.7%)	157 (38.8%)	​

### 3.2 Relationship between ADL and survival

**FIGURE 2:**
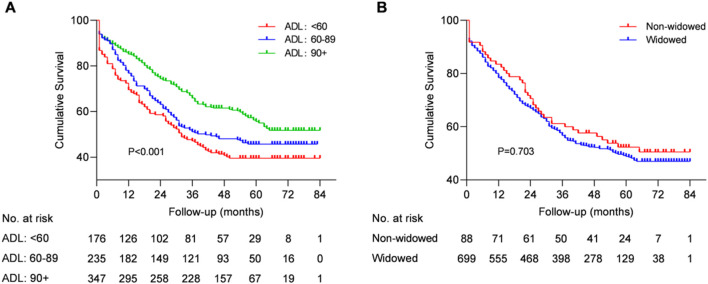
Kaplan-Meier survival curves and life tables stratified by ADL **(A)** and marital status **(B)**. The Log-rank test for the two predictors yielded *p* < 0.001 and *p* = 0.703, respectively.

Univariate and multivariate survival analyses adjusted for possible confounder variates of centenarians (Table 2; Figure 2A) revealed that ADL was a predictor of all-cause mortality in centenarians. A hazard ratio of 1.933 (95% CI: 1.411–2.648, *p* < 0.001) was found for those with ADL<60, while those with 60≤ADL<90 had a HR of1.438 (95% CI: 1.084–1.907, *p* = 0.012) when compared to centenarians with ADL≥90. The Lowess smooth curve was basically a horizontal straight line, suggesting that the above proportional hazards assumption was tenable (Supplementary Figure S1). Kaplan-Meier survival curves indicated that there was no difference between the two maritial status groups (Figure 2B).Therefore, the hypothese H1 was supported, while H2 was rejected.

**TABLE 2:** Cox proportional hazard model for predictors of survival among centenarians.

Models	ADL	HR (95% CI)	*p*
Model 1	60<	1.650 (1.290–2.110)	0.000
	60–89	1.345 (1.065–1.699)	0.013
	≥90	1	-
Model 2	60<	1.701 (1.327–2.180)	0.000
	60–89	1.389 (1.097–1.758)	0.006
	≥90	1	-
Model 3	60<	1.933 (1.411–2.648)	0.000
	60–89	1.438 (1.084–1.907)	0.012
	≥90	1	-

Model 1: Un-adjusted.

Model 2: Adjusted for age and gender.

Model 3: Adjusted for age, gender, maritial status, residential type, No. of children, No. of diagnoses and MMSE.

- Indicates no data.

### 3.3 Baseline characteristic of centenarians by marital status

Of the 787 centenarians, 699 (88.82%) were widowed (Table 3). Among the non-widowed centenarians, the proportion of males (42.05% vs. 15.45%), those with formal education (20.45% vs. 6.15), smokers (20.45% vs. 7.30), drinkers (19.32% vs. 9.01%) and those with severe cognitive impairment (12.50% vs. 5.15%) was significantly higher compared to the widowed group. However, there were no statistical differences between the two groups in terms of follow-up time, survival status, age, ethnicity, residential type, number of children, number of diagnoses, SBP, DBP, FBG, TC or TG.

**TABLE 3:** Profiles of centenarians grouped by marital status.

Variables	All (n = 787)	Non-widowed (n = 88, 11.2%)	Widowed (n = 699, 88.8%)	*p*
Survival time, mean (SD), month	54 (15, 60)	50 (20.25, 68)	54 (15, 60)	0.277
Survival status	​	​	​	0.771
Survivors	382 (48.5%)	44 (50.0%)	338 (48.4%)	​
Non-survivors	405 (51.5%)	44 (50.0%)	361 (51.6%)	​
Age, mean (SD), year	102 (101, 104)	102 (101, 104)	102 (101, 104)	0.489
Gender	​	​	​	0.000
Male	145 (18.4%)	37 (42.1%)	108 (15.4%)	​
Female	642 (81.6%)	51 (57.9%)	591 (84.6%)	​
Education	​	​	​	0.000
Educated	61 (7.8%)	18 (20.4%)	43 (6.2%)	​
Uneducated	726 (92.2%)	70 (79.6%)	656 (93.8%)	​
Ethnicity	​	​	​	0.363
Han	702 (89.2%)	76 (86.4%)	626 (89.6%)	​
Minority	85 (10.8%)	12 (13.6%)	73 (10.4%)	​
Residential type	​	​	​	0.180
With family	670 (85.1%)	79 (89.8%)	591 (84.5%)	​
Live alone	108 (14.9%)	8 (10.2%)	100 (14.3%)	​
Smoke	​	​	​	0.000
Yes	69 (8.8%)	18 (20.4%)	51 (7.3%)	​
No	668 (91.2%)	64 (72.7%)	604 (86.4%)	​
Drink	​	​	​	0.003
Yes	80 (10.2%)	17 (19.3%)	63 (9.0%)	​
No	663 (84.2%)	67 (76.1%)	596 (85.3%)	​
No. of children, medium (IQR)	4 (3, 6)	5 (3, 7)	4 (3, 6)	0.054
No. of chronic diseases, medium (IQR)	1 (0, 2)	1 (0, 1)	1 (0, 2)	0.150
SBP, mean (SD), mmHg	151.56 (24.95)	148.45 (22.01)	151.95 (25.29)	0.226
DBP, mean (SD), mmHg	75.12 (13.03)	73.64 (12.43)	75.31 (13.10)	0.270
Fasting blood glucose, mean (SD), mmol/L	5.15 (1.47)	5.37 (1.72)	5.12 (1.43)	0.226
Total cholesterol, mean (SD), mmol/L	4.68 (1.04)	4.66 (1.06)	4.68 (1.03)	0.832
Triglyceride, mean (SD), mmol/L	1.18 (0.62)	1.12 (0.56)	1.18 (0.63)	0.350
MMSE	​	​	​	0.004
≤9	320 (40.7%)	28 (31.8%)	292 (41.8%)	​
10–20	203 (25.8%)	32 (36.4%)	272 (38.9%)	​
≥21	47 (6.0%)	11 (12.5%)	36 (5.2%)	​
ADL score, medium (IQR)	85 (60, 95)	85 (60, 95)	85 (60, 95)	0.981
ADL	​	​	​	0.808
60<	176 (22.4%)	18 (20.5%)	158 (22.6%)	​
60–89	235 (29.9%)	28 (31.8%)	207 (29.6%)	​
≥90	347 (44.1%)	36 (40.9%)	311 (44.5%)	​

### 3.4 Predictors of survival by marital status

**FIGURE 3:**
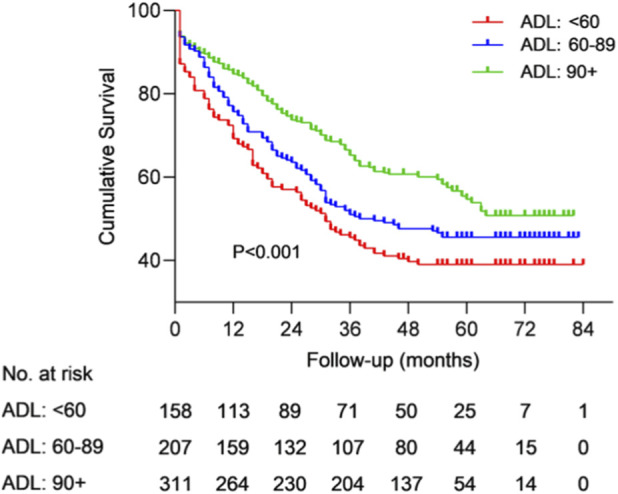
Kaplan-Meier survival curve and life table of widowed cantenarians. The Log-rank test was *p* < 0.001.

We stratified all centenarians into two marital groups for Cox proportional hazard regression analyses. The results revealed that ADL was significantly associated with survival in widowed centenarians (for ADL<60 vs. ADL≥90, HR = 2.020, 95% CI: 1.450–2.814, *p* < 0.001; for 60≤ADL<90 vs. ADL≥90, HR = 1.493, 95% CI: 1.108–2.011, *p* = 0.008), but not in non-widowed centenarians (all *p* > 0.05) across Model 1, Model 2 and Model 3. (Table 4; Figure 3). There was a decreasing trend in the HR within the ADL subgroups of widowed centenarians (all *p* > 0.05). The Lowess smooth curves were basically a horizontal straight line, suggesting that the above proportional hazards assumption were tenable (Supplementary Figures S2, S3). Therefore, the hypothese H3 was supported.

**TABLE 4:** Cox proportional hazard model for physical function of survival with different marital status.

Models	ADL	Non-widowed			Widowed		
		HR (95% CI)	*p*	*p* for trend	HR (95% CI)	*p*	*p* for trend
Model 1	60<	1.546 (0.693–3.446)	0.287	​	1.662 (1.283–2.152)	0.000	​
	60–89	1.497 (0.731–3.067)	0.270	​	1.330 (1.039–1.703)	0.024	​
	≥90	1	-	0.444	1	-	0.000
Model 2	60<	1.841 (0.773–4.385)	0.168	​	1.694 (1.307–2.197)	0.000	​
	60–89	1.610 (0.779–3.014)	0.198	​	1.373 (1.069–1.762)	0.013	​
	≥90	1	-	0.296	1	-	0.000
Model 3	60<	1.641 (0.528–5.098)	0.392	​	2.020 (1.450–2.814)	0.000	​
	60–89	0.879 (0.359–2.151)	0.778	​	1.493 (1.108–2.011)	0.008	​
	≥90	1	-	0.568	1	-	0.000

Model 1: Un-adjusted.

Model 2: Adjusted for age and gender.

Model 3: Adjusted for age, gender, residential type, No. of children, No. of diagnoses and MMSE.

- Indicates no data.

## 4 Discussion

Physiologically, as individuals age, they experience a range of challenges, including physical decline, the development of chronic diseases and social changes, such as children leaving home and the death of a spouse, along with reduced social engagement (Sheftel et al., 2024; Beard et al., 2016; Christensen et al., 2009). These issues significantly diminish the quality of life, leading to an increase in nursing and medical costs, especially for centenarians (Rozzini, 2016). These factors ultimately influence the expected survival time of the elderly (Cheng et al., 2020). Even more concerning is the cumulative effect of these adverse factors, which can accumulate either individually or in combination over time (Zhao et al., 2020; Xu and Yang, 2022).

The physiological capacity of the elderly declines progressively with age, or more rapidly due to diseases, accidents or other factors, resulting in disability or semi-disability of the elderly (Strax et al., 2010). The disability rate increases monotonously with age, meaning that as individuals grow older, the risk of becoming disabled rises (Lamarca et al., 2003; Chen et al., 2018). However, the age-related decline in ADL scores is well-documented (Chaoping et al., 2023). For example, the prevalence of disability has been reported to be 19% (95% CI: 17.8%–20.2%) in individuals over 60 years of age, and 36.85% of Europeans aged 71–80 years exhibit ADL impairment (Jitapunkul et al., 2003; Ćwirlej-Sozańska et al., 2018). According to the European Health Survey (Carmona-Torres et al., 2019), the prevalence of ADL impairment in those aged 85 years and older was 34%. In this study, the rate of disabled and semi-disabled centenarians was found to be 52.22%, significantly higher than in younger populations. These findings were consistent with those from the Danish 1895-West and 1915-West Birth Cohort Studies (1895-West vs. 1905-West: 53% vs. 50%) (Rasmussen et al., 2018). For centenarians, the compression of disability and morbidity toward the end of life is characteristic of their longevity (Terry et al., 2008).

Disability is well-known predictor of death, and disability in the elderly is often irreversible (Rupp and Dushi, 2017; Kuper et al., 2024). Not only does disability caused by diseases accelerate the death process, but the physiological disability also negatively impacts life expectancy and survival rates (Thomas and Barnes, 2010). A study (Wu et al., 2016) based on the National Health and Nutrition Examination Survey (NHANES) showed that disability increases all-cause mortality in older adults 60–84 years of age. Furthermore, physical activity was identified as a risk predictor of death for community-dwelling residents aged 75–83 years old (Male: HR = 1.65, 95% CI: 1.27–2.14; Female: HR = 1.77, 95% CI: 1.42–2.19) (Schultz-Larsen et al., 2012). A survey of older adults aged over 65 years in Taiwan revealed that 93% of older adults showed no ADL impairment 10 years before death (Chiu et al., 2021). However, as death approached, the prevalence of ADL impairments increased, with an average of 10% participants showing more than three ADL abnormalities 6 years before death, and moderate to severe disability affecting 38% of individuals in the year before death. The loss of physical activity, as demonstrated in this study, was associated with the mortality in centenarians. Additionally, a linear relationship was observed between physical function loss and mortality in centenarians.

The proportion of elderly individuals who are widowed increases with age. Among individuals aged 65 and older, 13% of men and 40% of women are widowed. This rate rises to 57% for those over 75 years old (Sundström et al., 2014). In this study, the proportion of centenarians without a partner reached 88.82%. Health deficits associated with bereavement may manifest earlier during the marital transition than previously anticipated, highlighting the need for attention to the health of older adults whose spouses are suffering chronic or life-limiting illness (Williams et al., 2008). The Longitudinal Study of Aging (1984–1990) revealed that marital status was associated with the health and survival of the elderly, with widowed men at higher risk of disability compared to non-widowed individuals (Goldman et al., 1982). Additionally, a health survey (Barrenetxea et al., 2023) conducted with 15,858 Singaporean Chinese aged 61–96 showed that widowed adults had a higher risk of death, a phenomenon often referred to as the Widowhood Effect (Zhu et al., 2022; Ennis and Majid, 2021). However, the preliminary analysis of this study found that marital status alone did not affect the mortality of centenarians.

Further stratified analysis found that the loss of physical capacity was not associated with mortality among non-widowed centenarians, which may be due to the limited simple size. On the other hand, physical disability was associated with high mortility among widowed centenarians. The reasons behind this phenomenon are multifactorial, encompassing changes in biological, social, and psychological factors (Robards et al., 2012; Cornwell and Qu, 2024). For the elderly, widowhood often results in living alone, which is a known risk factor for mental health issues and can increase the likelihood of death (Yang and Gu, 2021; Chen et al., 2022; Srivastava et al., 2021). Emotional support during the first 6 months after the loss of a spouse has been shown to improve survival rates (Penninx et al., 1997). Results from the Midlife in the United States cross-sectional survey (Yuan et al., 2024) showed that the greatest improvement in physical function occurred during the transition from widowhood to non-widowhood. This underscores the vital role that marriage, as a fundamental form of social support, plays in the health of the elderly (Rodin et al., 2024). After eliminated the influence of confounding factors such as living arrangements (living with or without family members), the possiblility of external help (number of children), and the possiblility of cognitive impairment (MMSE) on survival analysis, the above results were still valid. For some centenarians with poor economic conditions, the loss of spouse means that they will not be able to handle basic daily activities. Despite external supports given by their children, guardians or caregivers, some centenarians tend to be more adaptive to the care and companionship mode of their partners (Lau et al., 2024).

This study has several limitations. Firstly, the CHCCS cohort is based exclusively on centenarians from Hainan Island, China, which is geographically isolated from other regions. As a result, the findings may not fully represent the real situation in other regions. Secondly, due to the extended duration of one-by-one surveys, some centenarians had passed away before the survey was completed. It is possible that these deceased centenarians had poor ADL scores, which could affect the accuracy of the ADL, mortality, and survival data. Thirdly, excluded participants with missing baseline data may limit generalizability, and introduce bias to the analysis. Lastly, centenarians prone to make female survival, so potential gender differences in centenarians may bias or dilute the estimated associations. Future research should focus on the influence of gender differences on survival.

## 5 Conclusion

This study suggested that physically disabiled centenarians exhibited the highest mortility, while physically healthy centenarians demonstrated the highest survival rate. Further survival analysis revealed that physical disability and widowhood were both predictive factors influencing the survival of centenarians. Our findings suggest that, when a centenarian was physically disabled, or lost a spouse, caregivers and social workers should prioritize the needs of the vulnerable centenarians, implementing targeted interventions to improve their quality of life.

## Data Availability

The original contributions presented in the study are included in the article/Supplementary Material, further inquiries can be directed to the corresponding authors.
