# Supplementary figures and images for: Effects of physical disability and widowhood on the survival of centenarians: a 7-year follow-up of CHCCS centenarians
Source: Front Aging. 2026 Feb 24;7:1764198. doi: 10.3389/fragi.2026.1764198 (PMC12971909; doi:10.3389/fragi.2026.1764198)

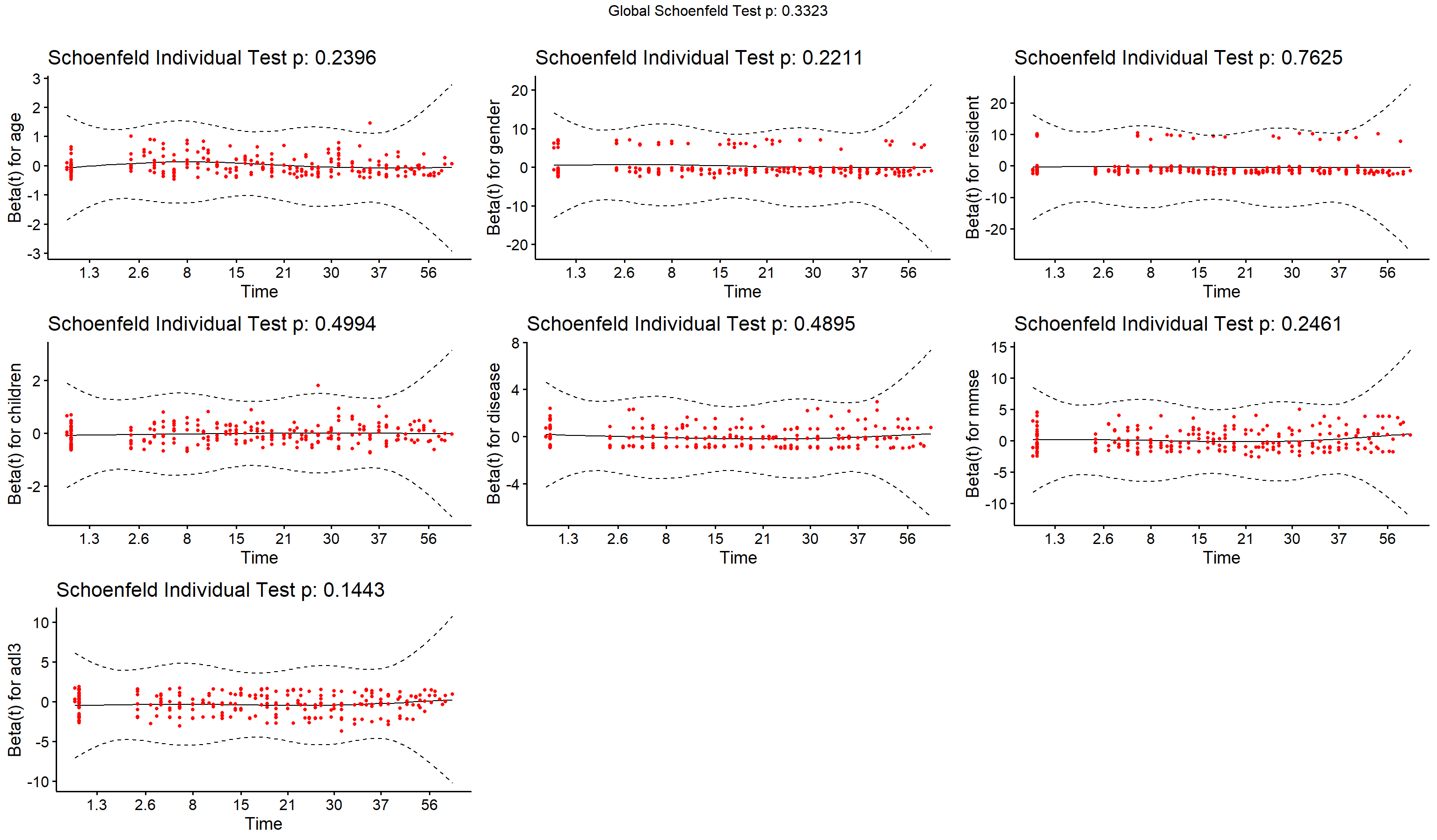

Supplement: Supplementary file 1 [file Image3.tif]

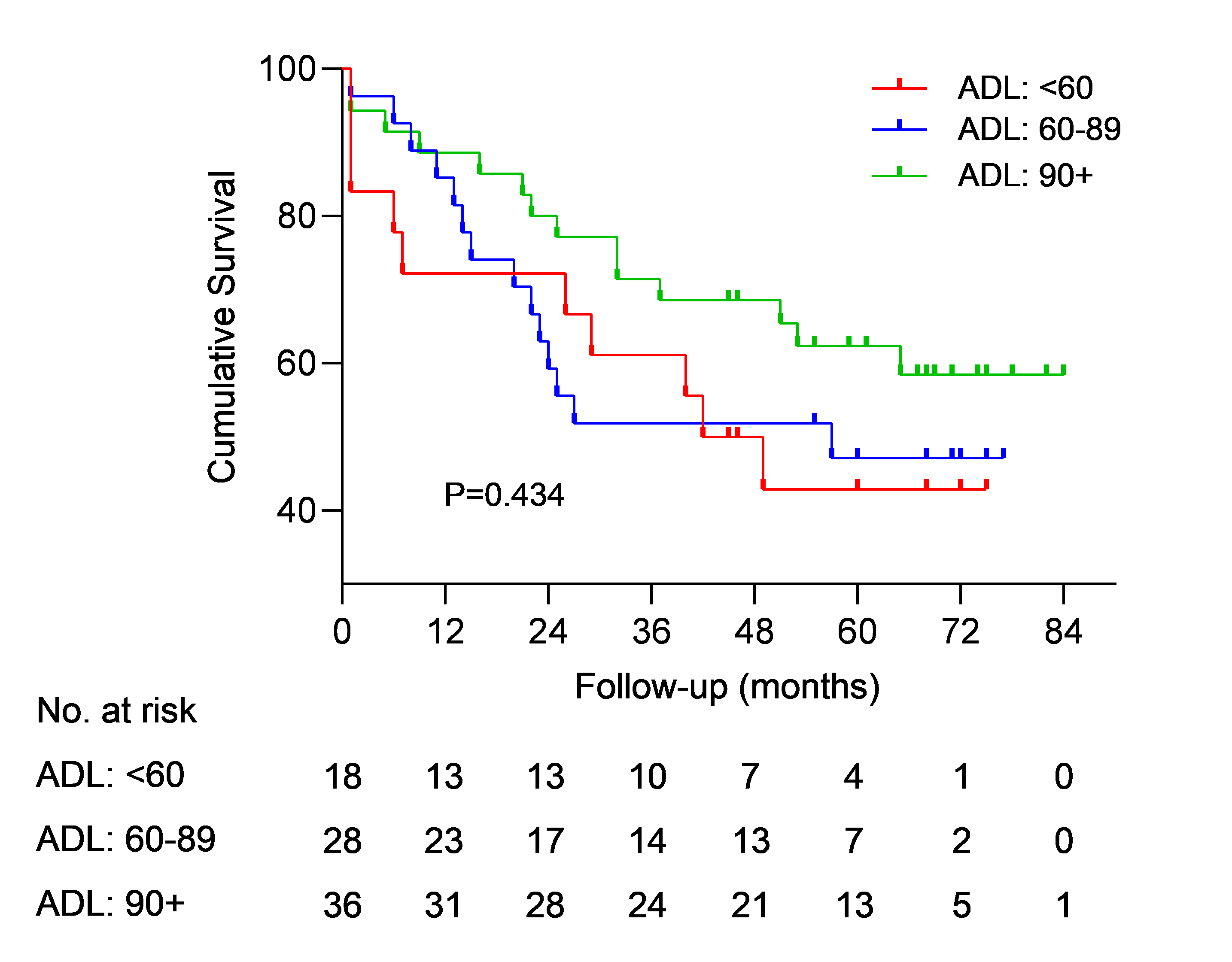

Supplement: Supplementary file 2 [file Image4.tif]

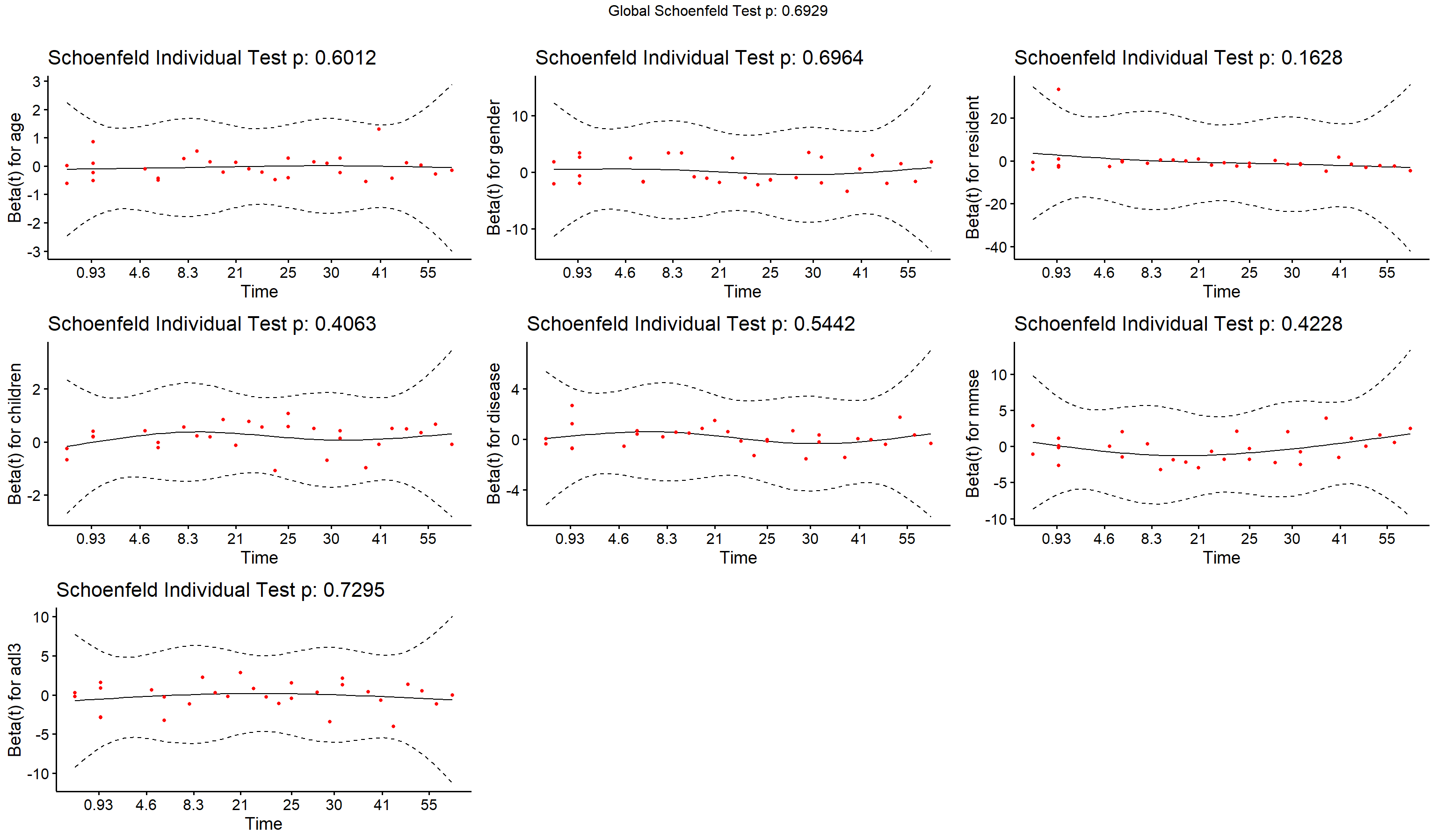

Supplement: Supplementary file 3 [file Image2.tif]

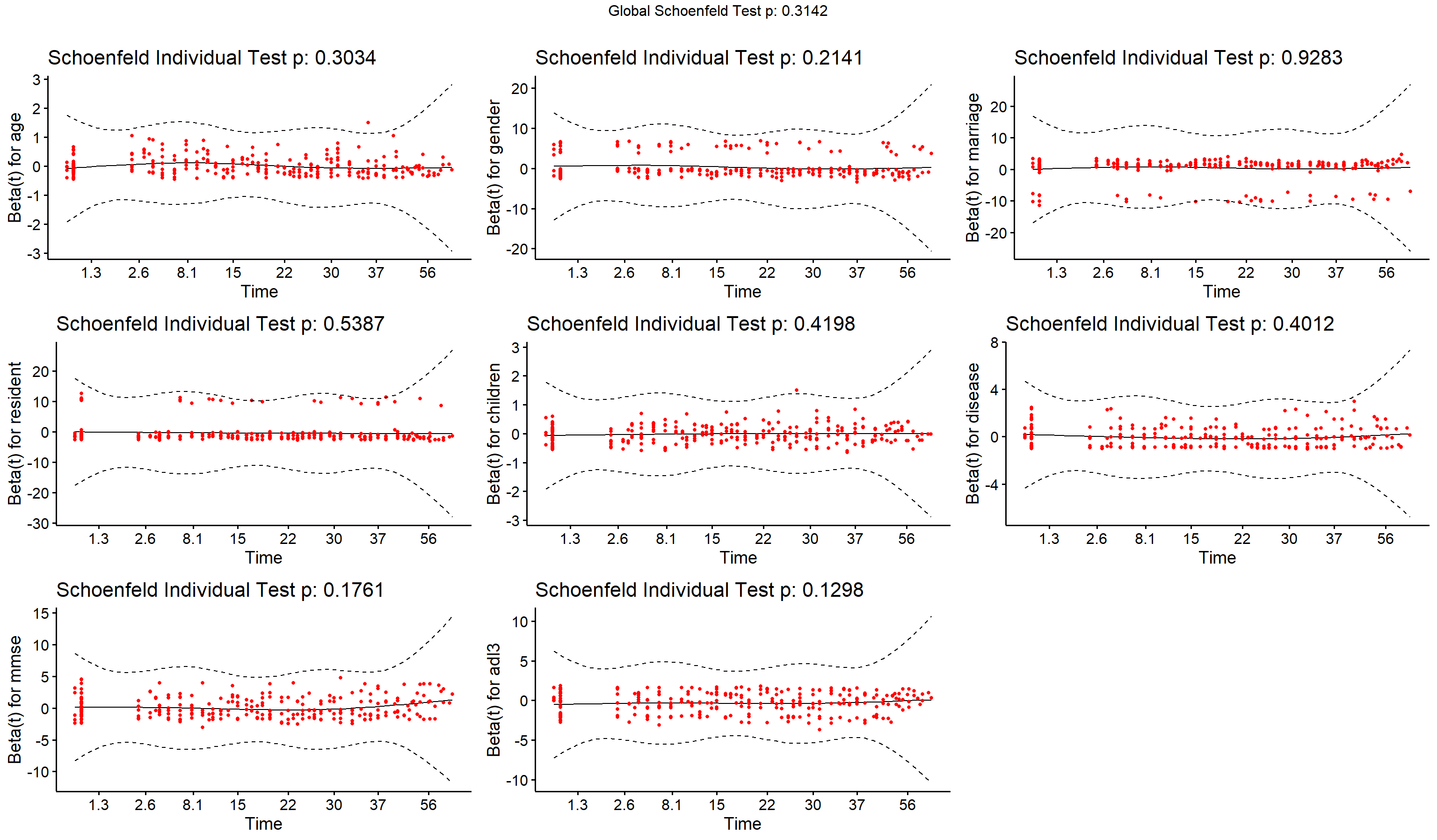

Supplement: Supplementary file 4 [file Image1.tif]
